# Plexin B2 tissue expression and related gene polymorphisms in psoriasis and their relation to NB-UVB and Acitretin therapy

**DOI:** 10.1007/s00403-024-02880-x

**Published:** 2024-05-11

**Authors:** Eisa Mohamed Hegazy, Moustafa A. El Taieb, Mohammed H. Hassan, Ahmed K. Ibrahim, Ebtehal A. El-Din, Hassan M. Ibrahim

**Affiliations:** 1 Dermatology, Venereology and Andrology Department, Qena Faculty of Medicine, South Valley University, Qena, Egypt; 2 Dermatology, Venereology and Andrology Department, Faculty of Medicine, Aswan University, Aswan, Egypt; 3 Medical Biochemistry Department, Faculty of Medicine, South Valley University, Qena, Egypt; 4 Community Medicine Department, Asuit University, Asuit, Egypt

**Keywords:** Psoriasis vulgaris, Plexin-b2, Real time PCR, SIRPγ-rs71212732, PASI, NB-UVB, Acitretin

## Abstract

Psoriasis is a chronic, immune-mediated, hyperproliferative skin disease. Etiopathogenesis of psoriasis is not well understood. Plexin B2 was found to have effects on CD100-mediated T-cell morphology and expressed in the immune system. It may play a role in the pathogenesis of psoriasis. To assess the tissue level of plexin-B2 and plexin B2 related gene polymorphism which is signal regulatory protein gamma (SIRPγ-rs71212732) in psoriatic patients before and after NB-UVB, acitretin therapy alone or in combination and to detect correlation between level of tissue plexin B2 and disease severity and improvement. This single blinded randomized controlled trial was carried on 50 psoriatic patients and 50 healthy controls. Psoriasis Area and Severity Index score (PASI) was used to evaluate the disease severity. Tissue plexin-b2 level was measured using ELISA and SIRPγ-rs71212732 (T\C) was assessed using TaqMan™ assays and real-time PCR. A significant lower tissue plexin-B2 level was observed in control group (2.9 ± 0.6 pg/g) than cases (25.8 ± 2.8, pg/g) (p < 0.001). Also, a significantly higher tissue plexin-B2 level was observed in sever psoriasis (32.7 ± 3.8 pg/ml) in than moderate psoriasis (13.6 ± 2.1 pg/ml, p = 0.001). Tissue plexin B2 was positively correlated with diseases severity. Significantly higher (TC& TT) genotypes and mutant (C) allele among patients compared to the controls, p < 0.001 for all. Tissue plexin-b2 level was high in psoriasis vulgaris with positive correlation with disease severity and decreased after treatment. This may indicate a role of plexin-b2 in psoriasis vulgaris pathogenesis.

## Introduction

Psoriasis is a common chronic inflammatory skin disease which may induce itchy or painful lesions and negatively affect quality of life. Its prevalence is variable between 0.51% to 11.43% in different countries [1]. The exact aetiopathogenesis of psoriasis is not completely explained. Pathological mechanism involves skin inflammation and hyperproliferation of keratinocytes induced innate and adaptive immune cells. Genetic, immunological and environmental factors are considered the most important aetiologies [2]. Recently, keratinocytes are considered the keystone player in the pathogenesis of psoriasis [3]. Keratinocytes can lead to damage which activate the inflammation in psoriasis [4]. Plexins (PLXN) are receptors for semaphorins; they are a family of trans-membrane proteins act on epithelial repair process through its interaction with CD100. PlxinB2 has effects on CD100-mediated T-cell morphology [5]. Plexins are expressed in the immune system and cells inducing cell movement & interaction [6]. There is no complete curative therapy for psoriasis up till now, and available treatment is only to control disease activity, severity and improve symptoms [7]. Topical treatments including topical corticosteroids, vitamin D3 analogues, tar-based preparations, dithranol, salicylic acid and topical retinoids are safe and effective for mild to moderate disease. Systemic therapy, including phototherapy, acitretin, methotrexate, cyclosporine, or biologic therapy may be required for severe disease^7^. NB-UVB has beneficial effects on the course & quality of life by improvement of different oxidative stress parameters [8]. Several studies have been investigated the efficacy and safety of acitretin in psoriasis as a mono therapy or combined therapy& its side effects have been shown to be dose dependent & reversible after decreasing the dose [9]. Signal Regulatory Proteins (SIRPs) are classified as “paired receptors” since they show the following characteristics: (1) different genes encode SIRPS (2) they have similar significant sequence in their extracellular domains and (3) they have both activating and inhibitory members [10, 11]. Signal Regulatory Protein γ (SIRPγ) is a three cell surface receptors implicated in modulating immune responses. SIRPγ is expressed on T lymphocytes & lead to adhesion of lymphocytes to antigen-presenting cells [11]. SIRPγ resulting in increased cell–cell adhesion in an integrin-independent manner as it expressed by T cells where it interacts with CD47 on the surface of the cell [11, 12].

## Patients and methods

This single blinded randomized controlled trial was carried out on 50 patients diagnosed as psoriasis vulgaris and 50 age, sex and BMI matched normal subjects selected as a control group. The study was carried out at the outpatient Dermatology Clinic of Qena South Valley University Hospitals, Qena, Egypt in the period between December 2021 and March 2023.

Sample size calculation was carried out using G*Power 3 software [13]. A calculated minimum sample of 96 respondents (Group I (n = 48); cases with psoriasis vulgaris and Group II (n = 48) age/sex/BMI matched control to detect an effect size of 0.75 [14] in the mean Plexin-b2 level with an error probability of 0.05 and 95% power on a two-tailed test.

Pregnant/lactating females, females willing to be pregnant in the next 3 years, those with hyperlipidemia, history of any inflammatory diseases (atopic dermatitis, asthma, ulcerative colitis and Crohn’s disease and any other similar disease), patients received NB-UVB phototherapy or acitretin in the last 6 months, patients treated with methotrexate or biologic agents and any systemic treatment of psoriasis were excluded from the present study.

Patients were randomly assigned by using coded cards into 3 groups:

*Group 1:* included 17 patients treated with acitretin in dose of 0.75 mg per kg per day orally for 3 months.

*Group 2:* included17 patients treated with NB-UVB phototherapy three sessions weekly for three months with maximum dose of 1400 mJ/cm2. Dosing for NB-UVB was based on the Fitzpatrick skin phototype and the minimal erythema dose to NB-UVB (MED-B). The starting dose was 00.20 mJ based on skin phototype, or 70% of the MED-B to avoid erythema. The dose is subsequently increased by 10–15% per session. Sessions were given three times weekly non-consecutive days for three months using (WaldmannUV5000 cabinet equipped with WaldmannF85/100W-UV01 tubes, EU).

*Group 3* included 16 patients treated with acitretin in dose of 0.75 mg per kg per day orally plus NB-UVB phototherapy three sessions weekly for three months with the same dosing of group 2.

### Ethical considerations

The study was approved by the Institutional review board (IRB) of the Faculty of Medicine-Qena University prior to study execution. Ethical approval code: SVU, MED, DVA012, 2, 218, 226. The study was registered at clinical trial.com. Approval number: NCT05184348.

All participants received a written consent form. The informed consent was clear and indicated the purpose of the study, and their freedom to participate or withdraw at any time without any obligation. The form also indicated the agreement or rejection of participants to publish data including patient photos. Furthermore, participants’ confidentiality and anonymity were assured by assigning each participant with a code number for the purpose of analysis only. The study was not based on any incentives or rewards for the participants. The study was in line with the Declaration of Helsinki.

### Clinical assessment

General examination was done to exclude any associated systemic illness and any similar disease. Local examination was done to evaluate the severity of psoriasis vulgaris. The Psoriasis Area and Severity Index score (PASI) was calculated for all patients & categorized as mild (< 10) or moderate to severe (≥ 10)^15^, then venous blood sample 2 ml were drained to determine plexin-b2 related gene polymorphism & skin biopsy from psoriasis lesions in patients and from healthy skin of the volunteers were taken for assay of tissue plexin-b2.

### Tissue Plexin-B2 assays

Skin biopsies were taken from psoriasis lesions of patients and from healthy skin of volunteers by 3 ml punch biopsy following local anaesthesia using lidocaine 2%. The skin biopsies were homogenized using lysis buffer (Tris–HCL). The buffer contain S1%protease inhibitor cocktail (cell signalling technology inc, Danavers, MA,USA).

The homogenizer (glass/Teflon homogenizer), and then homogenates were stored frozen at − 80 °C till time of plexin B2 assay using commercial available ELISA kit supplied by Chongqing Biopsies co, China with catalog No: BZEK1453-96 based on standard sandwich enzyme-linked immune-sorbent assay technology. The purified anti- plexin B2 antibody was pre-coated onto 48-well plates and the HRP conjugated anti-PLXNB2 antibody was used as detection antibodies.

Tissue level of plexin B2 were determined by using microplate ELISA reader (EMR-500, USA).The human plexin B2 concentration of the samples was interpolated after spectrophotometric determination of total protein content (using commercial kits supplied by Spectrum Diagnostics, Egypt, cat no 310001) of each tissue homogenate sample with a spectrophotometer (Chem-7, Erba Diagnostics Mannheim GmbH, Made in Germany) as the ratio of the plexin B2 concentration to each gram of tissue proteins was calculated.

### Genetic assay of plexin b2 related gene polymorphism ( signal regulatory protein gamma) SIRPγ-rs71212732)

**Fig. 1:**
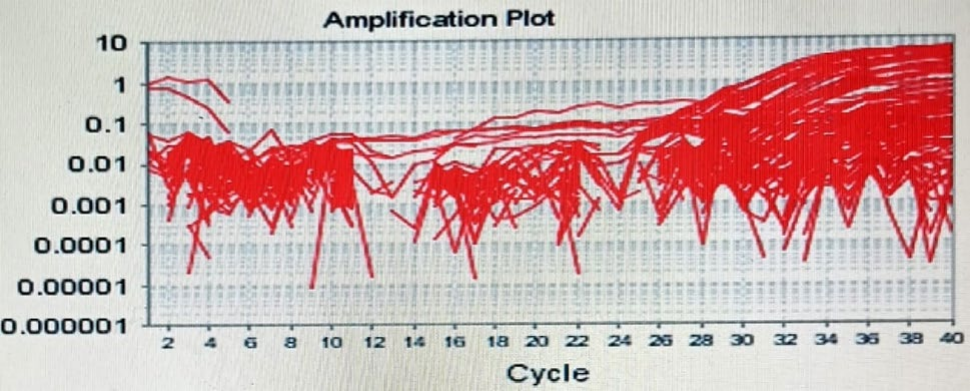
Amplification plot of rs71212732

**Fig. 2:**
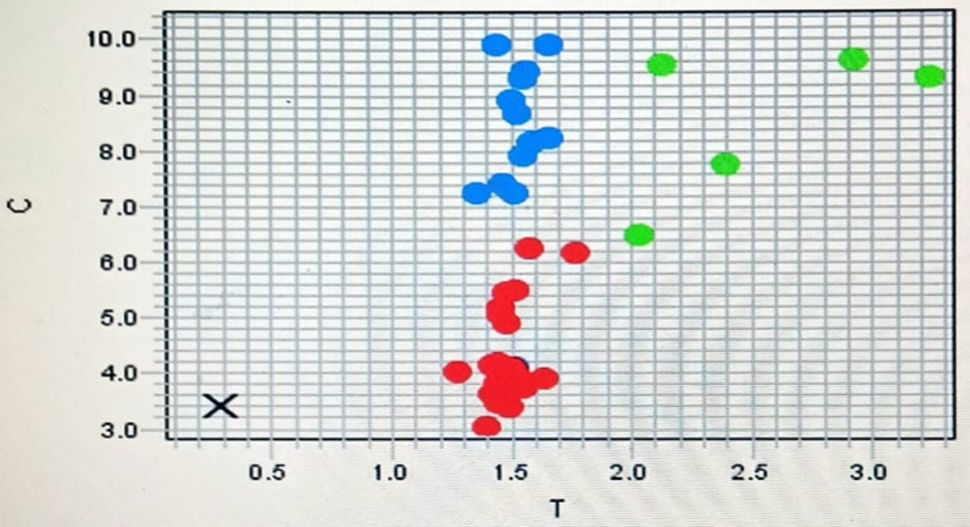
Allelic discrimination plot of rs71212732

Firstly, the genomic DNA was extracted from each stored whole EDTA blood sample using Pure Link™ Genomic DNA Mini Kit Catalog No. K1820-00, inverter US, according to the manufacture protocol. Genotyping of DNA samples were performed by QRTPCR: 7500 fast real time PCR, Applied Biosystem, USA., (VIC/FAM). CAGCTGAGCAAATCAAAAGTGACA[T/C]TCTTCTTAGATCTGTCAGAAAAAC specifically designed to distinguish the variant of T/C (rs71212732) TaqMan genotyping Master Mix (20 ul) cat. No. (4,371,355) 2 × 10 ul, DNA (10 ng /ul) 2 ul, Primer and probe assay 30 × cat. No. (4,351,379) C_167071991_10 0.7 ul, RNase free water 7.3 ul. Hot start step at 95 °C for 7 min, initial denaturation for 20 s at 95°c, annealing and extension for 60 s at 59°c, in 40 cycles, using amplification and alleles discrimination plots (Fig. 1,2).

### Statistical analysis

Statistical analysis was conducted using IBM-SPSS ver. 24, categorical variables were presented as frequency and percentages, and comparison of proportions between groups was conducted using Chi-square test. Quantitative variables were presented as mean, median, standard deviation (SD), and range. Comparison of quantitative data was conducted using Mann Whitney U test after normality testing using Shapiro–Wilk test. For continuous variables with more than two categories; ANOVA test was calculated to test the mean differences of the data that follow normal distribution and independent, post-hoc test was calculated using Bonferroni corrections. For repeated measure (pre- vs, post-treatment); paired sample t-test was used. Student t-test was calculated to test the mean differences in continuous variables between groups. Multivariate logistic regression analysis was calculated to investigate the effect of gene expression on the disease parameters (Odds Ratio—OR-, 95% confidence interval—95% CI- and p-value-). A p-value < 0.05 was considered significant, sample size calculated according to:

$$n(each\;group) = \frac{{(p_0 q_0 + p_1 q_1 )(z_{1 - \alpha /2} + z_{1 - \beta } )^2 }}{(p_1 - p_0 )^2 }$$

The studied SNP followed Hardy–Weinberg equation.

## Results

At the baseline of the study, age, sex, BMI distribution was comparable between the study groups with no significant difference as shown in (Table 1).

**Table 1:** Baseline Demographic Characteristics of the studied groups

	Control (1) (n = 50)	NB-UVB (2) (n = 17)	Acitretin (3) (n = 17)	Combined (4) (n = 16)	P-value
Age/years					0.272*
Mean ± SD	41.37 ± 12.1	44.41 ± 13.5	47.47 ± 14.4	46.25 ± 13.7	
P-value**	1 vs 2 = 0.407	2 vs 3 = 0.494	3 vs 4 = 0.874	1 vs 4 = 0.153	
	1 vs 3 = 0.098	2 vs 4 = 0.607			
Sex					0.369***
Male	30 (60%)	10 (58.8%)	12 (70.6%)	8 (50%)	
Female	20 (40%)	7 (41.2%)	5 (29.4%)	8 (50%)	
BMI					0.776*
Mean ± SD	28.31 ± 4.4	27.38 ± 2.7	27.91 ± 2.6	28.56 ± 2.9	
P-value**	1 vs 2 = 0.371	2 vs 3 = 0.675	3 vs 4 = 0.612	1 vs 4 = 0.811	
	1 vs 3 = 0.700	2 vs 4 = 0.359			

*ANOVA test was used to compare the mean difference between groups

**Post-hoc test with Bonferroni Corrections was used to compare the mean difference between groups

***Chi-square test was used to compare frequency between groups

Regarding disease characteristics, No statistically significant difference was observed regarding disease duration between the studied groups duration (p = 0.521). BMI also showed no statistically significant difference between study groups (p = 0.477). The rate of comorbidity (p = 0.293), the source of lesion, and type of lesions (p = 0.903) showed no significant difference as shown in (Table 2).

**Table 2:** Comparison of Disease Characteristics among the studied cases

	NB-UVB (1) (n = 17)	Acitretin (2) (n = 17)	Combined (3) (n = 16)	P-value
Disease duration/years				
Mean ± SD	7.72 ± 1.7	10.26 ± 2.2	10.56 ± 1.8	0.521*
P-value**	1 vs 2 = 0.350	2 vs 3 = 0.914	1 vs 3 = 0.305	
BMI				
Mean ± SD	27.38 ± 2.7	27.91 ± 2.6	28.56 ± 2.9	0.477*
P-value**	1 vs 2 = 0.579	2 vs 3 = 0.503	1 vs 3 = 0.227	
Comorbidity				0.293***
No	8 (47.1%)	8 (47.1%)	11 (68.8%)	
HTN	6 (35.3%)	7 (41.2%)	3 (18.8%)	
DM	3 (17.6%)	2 (11.7%)	2 (12.4%)	
Lesion type				
Primary	4 (23.5%)	3 (16.7%)	3 (18.8%)	0.730***
Recurrent	13 (76.5%)	14 (82.4%)	13 (81.3%)	0.903***
Disease severity				
Moderate	6 (35.3%)	5 (29.4%)	7 (43.8%)	0.690
Severe	11 (64.7%)	12 (70.6%)	9 (56.2%)	
Improvement				
Mild	12 (70.6%)	5 (29.4%)	0 (0%)	
Moderate	5 (29.4%)	4 (23.5%)	6 (37.5%)	< 0.001
Marked	0 (0%)	8 (47.1%)	10 (62.5%)	

*ANOVA test was used to compare the mean difference between groups

**Post-hoc test with Bonferroni Corrections was used to compare the mean difference between groups

***Chi-square test was used to compare frequency between groups

At the start of the study, there was no significant difference in severity distribution among patient groups (p = 0.690). After treatment, a significant marked improvement was noticed in patients treated with combination therapy than each treatment alone (62.5%), patients treated with acitretin alone showed (47.1%) improvement, patients treated with NB-UVB alone showed (0%) improvement,with (p < 0.001), a significant moderate improvement was observed in patients treated with combination therapy (37.5%) than each treatment alone, patients treated with acitretin alone showed (23.5%) improvement, patients treated with NB-UVB alone showed (29.4%) improvement, with (p < 0.001) & mild improvement was observed in patients treated with NB-UVB alone showed (70.6%) improvement, patients treated with acitretin alone showed (29.4%) improvement, patients treated with combination alone showed (0) improvement, with (p < 0.001) as shown in (Table 2).

In the present study, tissue plexin-B2 level was significantly lower in control group (2.9 ± 0.6 pg/g) compared to cases (25.8 ± 2.8, pg/g) (p < 0.001). The differences in tissue plexin-B2 data between primary cases and recurrent cases showed a significantly higher mean was observed in primary cases (32.9 ± 7.3 pg/g tissue) compared to recurrent cases (24.1 ± 0.1 pg/g tissue) (p = 0.221) (Table 3).

**Table 3:** Differences in Plexin-B2 of the studied groups

	Tissue Plexin-B2 Level (pg/g tissue protein) (Mean ± SD)	P-value
Disease Status		< 0.001*
Control (n = 50)	2.92 ± 0.6	
Case (n = 50)	25.84 ± 2.8	
Recurrence		= 0.221*
Primary (n = 10)	32.86 ± 7.3	
Recurrent (n = 40)	24.08 ± 3.1	
Severity		
Moderate (n = 18)	13.71 ± 3.3	= 0.001*
Severe (n = 32)	32.71 ± 3.8	
Improvement		
Mild/Moderate (n = 32)	25.61 ± 4.1	= 0.916*
Marked (n = 18)	26.24 ± 3.4	
Treatment Effect		
Pre-treatment (n = 50)	25.84 ± 2.8	= 0.005**
Post-treatment (n = 50)	7.06 ± 1.5	

*Independent Sample t-test was used to compare the difference in mean between groups

**Paired Sample t-test was used to compare the difference in mean between groups

In our study tissue plexin-B2 was positively correlated with disease severity, mean tissue plexin-B2 was (32.7 ± 3.8 pg/ml) in severe cases in comparison with moderate cases (13.6 ± 2.1 pg/ml) with significant difference (p = 0.001) as showed in Table 3.

**Fig. 3:**
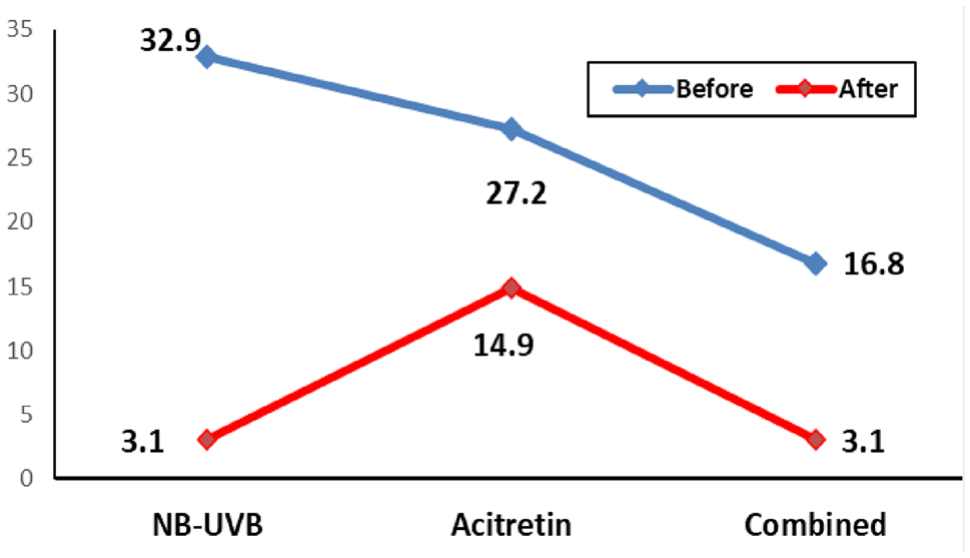
Effect of Treatment on the T. Plexin-B2 level

Tissue plexin-B2 level was significantly decreased after treatment in all cases i.e., baseline tissue plexin-B2 was (25.8 ± 2.8 pg/ml) and decreased to (7.06 ± 1.6) pg/ml) after therapy (p = 0.005). There was no significant difference in tissue plexin-B2 level according disease improvement after therapy between all cases, marked improvement was (26.2 ± 3.4 pg/ml) in comparison with mild to moderate improvement (25.6 ± 4.1 pg/ml) with non-significant difference (p = 0.916) as showed in (Table 3, Fig. 3).

The validity of the plexin-b2 level for prediction of disease severity is shown in Table 4. T.plexin-b2 level had a good predictive power for disease severity, AUC = **0.814**, p = ** < 0.001**; 95% CI: **0.695–0.933**. Moreover, at ≥ **12 pg/g tissue**, the validity criteria were as follows;** 78%**sensitivity, i.e., t. plexin-b2 correctly identified 78% of positive cases as having severe disease. Also, **73%** specificity, additionally, the test had 74% precision—Positive Predictive Value (PPV) i.e., the ability of the test to predict actively diseased patients among all positive cases. It also had 77% Negative Predictive Value (NPV) i.e., the ability to predict those with inactive disease among all those diagnosed as negative and overall, the test had 75.5% accuracy (Table 4).

**Table 4:** Diagnostic criteria of Pre-treatment T. Plexin-b2 for Prediction of Disease Severity

Diagnostic criteria	Disease Severity	Improvement
AUC	0.814	0.701
95% CI	0.695–0.933	0.546–0.856
P-value***	< 0.001	= 0.020
Cutoff	≥ 12 pg/g tissue	≤ 4 pg/g tissue
Accuracy	75.5%	71%
Sensitivity%	78%	73%
Specificity%	73%	69%
PPV%	74%	70%
NPV%	77%	72%

Sensitivity (true positives/all diseased); specificity (true negatives/all non-diseased); PPV (true positives/all test positives); NPV (true negatives/all test negatives)

*AUC* Area under the Curve, *SE* Standard Error, *CI* Confidence Interval

***Null hypothesis: true area = 0.5

**Fig. 4:**
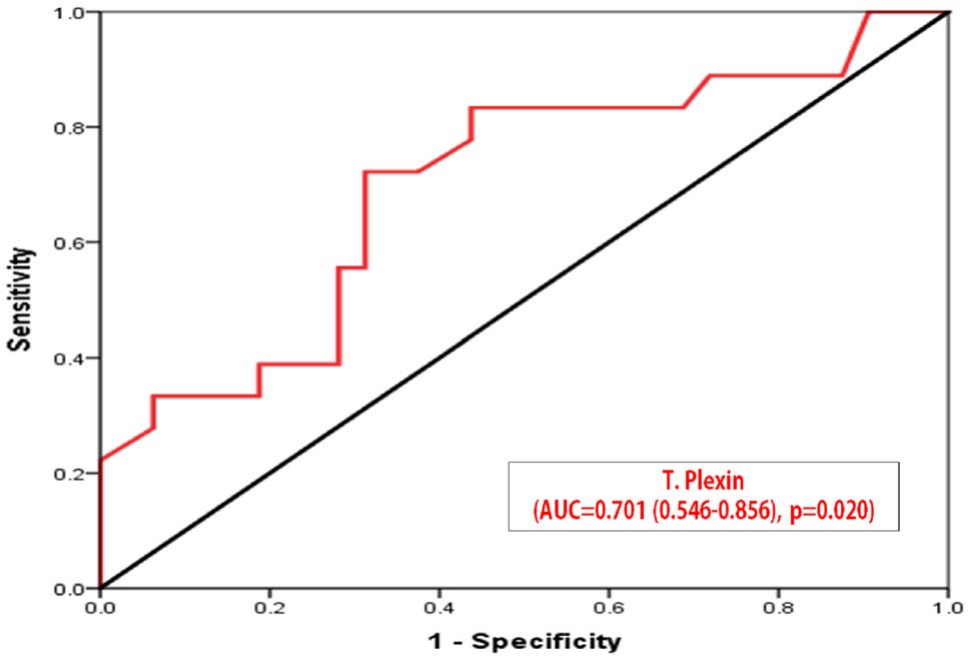
ROC Analysis of T. Plexin Level for Improvement Prediction

Regarding the effect of treatment on plexin-B2, a non-significant difference was observed in the mean tissue plexin-B2 level before treatment in study groups with mean level (32.9 ± 6.1 pg/g) in NB-UVB group compared to acitretin (27.2 ± 4.7 pg/g, p = 0.390) and combined group (16.8 ± 2.3 pg/g). (p = 0.062). Likewise, a significant difference was observed in the mean tissue plexin-B2 level after treatment with mean values (14.9 ± 3.9 pg/g) for acitretin group compared to NB-UVB (3.1 ± 0.6, pg/g) and combined group (3.1 ± 0.4) (p < 0.001). There was significant reduction in the tissue plexin-B2 level after treatment for the three groups (< 0.001) (Fig. 4).

**Fig. 5:**
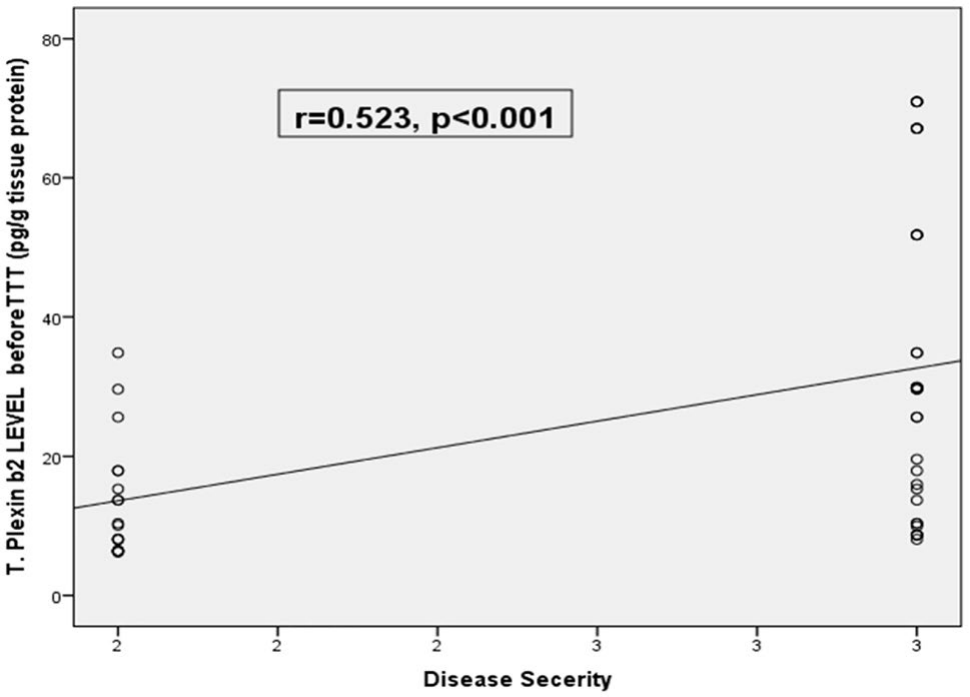
Correlation between Pre-treatment T. Plexin-b2Level and Disease Severity

Tissue plexin-b2 level had a good predictive power for marked improvement, AUC = 0.701, p = 0.020; 95% CI: 0.546—0.856. Moreover, at 4 pg/g tissue, the validity criteria were as follows; 73% sensitivity, i.e., tissue plexin-b2 was correctly identified 73% of positive cases as having marked improvement. Also, 69% specificity, the test correctly identified 69% of those without marked improvement as negative. Additionally, the test had 70% precision positive predictive value (PPV) i.e., the ability of the test to predict actively diseased patients among all positive cases. It also had 72% negative Predictive Value (NPV) i.e., the ability to predict those with inactive disease among all those diagnosed as negative and overall, the test had 71% accuracy as showed in (Fig. 5).

Genotype and allele Frequencies of SIRPγ-rs71212732 (T\C) SNP in studied cohort are illustrated in Table 5. For the genotype frequency distribution (TT/TC/CC), there was significant difference between cases and controls (p < 0.001). Compared with wild homozygous (TT), cases had 10.2 (3.2–13.8) times mutant heterozygous (TC) and 48.5 (27.9–64.7) times mutant homozygous (CC). For the genotype frequency distribution (TT + TC/CC), there was significant difference between cases and controls (p < 0.001). Compared with TT + TC, cases had 19.5 (9.4–27.5) times mutant homozygous (CC). Likely, for the genotype frequency distribution (TT/TC + CC), there was significant difference between cases and controls (p < 0.001). Compared with wild homozygous (TT), cases had 34.7 (20.9–44.8) times CC + TC. For the allele distribution (T/C), there was significant difference between cases and controls (p < 0.001). Compared with T-allele, C-allele had 16.9 (10.5–22.7) times among cases. There were insignificant differences in the association between genotype and allele Frequencies of rs71212732 (T\C) polymorphism and both disease severity and showed the differences in the Plexin-B2 data of the studied cohort. For the Plexin-B2 Genotype, the four groups showed significant difference (p < 0.001) i.e., the majority of control group (90%) had wild homozygous gene (TT) with only 10% (n = 5) had mutant heterozygous gene (TC). Contrarily, in the NB-UVB group: about one-quarter had wild homozygous gene (TT), 41% had mutant heterozygous gene (TC) and about one-third had mutant homozygous gene (CC) (Table 5).

**Table 5:** Genotypes and allele Frequencies of signal regulatory protein gamma rs71212732 (T\C) single nucleotide polymorphism in Cases vs. Control/Disease Severity/Disease Improvement

Group	SIRPG rs71212732 (T\C)								
	Genotypes							Alleles	
	TT	TC	CC	TT + TC	CC	TT	TC + CC	T	C
Case	15 (30%)	17 (34%)	18 (36%)	32 (64%)	18 (36%)	15 (30%)	35 (70%)	47 (47%)	53 (53%)
Control	45 (90%)	3 (6%)	2 (4%)	48 (96%)	2 (4%)	45 (90%)	5 (10%)	93 (93%)	7 (7%)
P-value	< 0.001			< 0.001		< 0.001		< 0.001	
OR (95% CI)				19.5 (9.4–27.5)		34.7 (20.9–44.8)		16.9 (10.5–22.7)	
Severity									
Moderate	9 (60%)	11 (64.7%)	12 (66.7%)	20 (62.5%)	12 (66.7%)	9 (60%)	23 (65.7%)	29 (29%)	35 (35%)
Sever	6 (40%)	6 (35.3%)	6 (33.3%)	12 (37.5%)	6 (33.3%)	6 (40%)	12 (34.3%)	18 (18%)	18 (18%)
P-value	= 0.697			= 0.508		= 0.470		= 0.514	
OR (95% CI)				1.2 (0.4–4.1)		1.3 (0.4–4.4)		1.3 (0.3–5.5)	
Improvement									
Mild/Mod	8 (53%)	14 (82.4%)	10 (55.6%)	22 (68.8%)	10 (55.6%)	8 (53%)	24 (68.6%)	30 (30%)	34 (34%)
Marked	7 (47%)	3 (11.6%)	8 (44.4%)	10 (31.2%)	8 (44.4%)	7 (47%)	11 (31.4%)	17 (17%)	19 (19%)
P-value	= 0.151			= 0.246		= 0.304		= 0.957	
OR (95% CI)				.8 (0.5–5.8)		0.5 (0.2–1.8)		1.1 (0.4–4.8)	

Table 6 showed the multivariable logistic regression model of the predictors of marked response. Regarding tissue plexin, with every pg/g tissue increase, there was 11% decrease (OR = 1.106, 95% CI: 1.001–1.224) in the chance of marked response and it was statistically significant (p = 0.049). There was insignificant negative/positive minimal to mild correlation with age, sex, and BMI and disease duration. Unlikely, there was significant (< 0.001) positive high and high moderate correlation between tissue plexin-b2 and disease severity (r = 0.523). In other words, increase in the level of tissue plexin-B2 was associated with higher disease severity (Table 6). according treatment group, cases in the combined group had 2.7 times (OR = 2.7, 95% CI; 1.02–4.62) more liable for marked response than NB-UVB group patients and it was statistically insignificant (p = 0.043).

**Table 6:** Independent predictors of response: multivariable logistic regression model

	OR (95% CI)	P-value
Age/years	0.971 (0.929 – 1.015)	= 0.192
Sex (Male)	1.556 (0.467 – 5.182)	= 0.472
BMI	0.967 (0.782 – 1.194)	= 0.752
Primary vs recurrence	1.238 (0.299 – 5.143)	= 0.769
Comorbidity	1.034 (0.454 – 2.289)	= 0.935
DD/days	0.975 (0.902 – 1.054)	= 0.520
Disease severity (Severe)	1.779 (0.510 – 6.207)	= 0.366
T. Plexin	1.106 (1.001 – 1.224)	= 0.049
CC Group	1.760 (0.534 – 5.802)	= 0.353
C-allele	0.524 (0.152 – 1.811)	= 0.307
TT Group	1.909 (0.552 – 6.599)	= 0.307
T-allele	1.760 (0.534 – 5.802)	= 0.353

*OR* odds ratio, *CI* confidence interval

## Discussion

Although the progress in the research on psoriasis in the last years, the exact aetiopathogenesis and the novel treatment are still missing. In the present study we aimed to put a hint on the possible role of plexin B2 in psoriasis pathogenesis and correlate it with treatment by acetritine and NB-UVB.

In our results, significantly lower tissue plexin-B2 level, was observed in control group compared to cases Significantly higher tissue plexin-B2 level, mean was observed in sever psoriasis in comparison with moderate psoriasis & tissue plexin-B2 level had a good predictive power for disease severity.

Hemida AS agreed with our study and found that was a significant positive correlation between plexin-B2 expression and psoriasis severity. There was a significant increased expression of plexin-B2 in proliferating keratinocytes from controls to peri-lesional (116 ± 41.95) and lesional (159.7 ± 63.05) skin (*P* < 0.001). Also, plexin-B2 showed significant expression in dermal inflammatory cells of lesional psoriatic skin (153.67 ± 72.71) when compared to control skin (25.71 ± 11.34) (*P* < 0.001). Plexin B2 could promote skin inflammation, as well as keratinocyte proliferation in psoriasis vulgaris; therefore, it may be used as a targeted therapy for psoriasis treatment [15].

In the present study, tissue plexin-B2 level showed a significant reduction in the level after treatment. Tissue higher percentage of marked improvement was observed in the combined group, then acitretin, with no cases in NB-UVB group. A higher percentage of moderate improvement was observed in the combined group then acitretin and NB-UVB group, so we advise use NB-UVB as Monotherapy in mild cases and acitretin alone in moderate cases but combined therapy by acitretin and NB-UVB in recalcitrant or sever cases. Lee& li, agreed with our study and found that acitretin is an effective systemic therapy for psoriasis and better to be combined with NB-UVB if not effective as Monotherapy [16].

Kampitak& Asawanonda agreed with our study and found that combination of low dose acitretin (25 mg/day) and NB-UVB was well tolerated and associated with typical retinoid and NB-UVB side effects and resulted in marked improvement of patients [17].

In our results, there was a significant difference between cases and controls in genotype frequency distribution (TT/TC/CC).Compared with wild homozygous (TT), cases had 10.2 (3.2 – 13.8) times mutant heterozygous (TC) and 48.5 (27.9 – 64.7) times mutant homozygous (CC). For the genotype frequency distribution (TT + TC/CC), there was a significant difference between cases and controls (p < 0.001). Compared with TT + TC, cases had 19.5 (9.4 – 27.5) times mutant homozygous (CC). Likely, for the genotype frequency distribution (TT/TC + CC), there was a significant difference between cases and controls (p < 0.001). Compared with wild homozygous (TT), cases had 34.7 (20.9 – 44.8) times CC + TC. For the allele distribution (T/C), there was a significant difference between cases and controls. Compared with T-allele, C-allele had 16.9 (10.5 – 22.7) times among cases and there were insignificant differences in the association between genotype and allele Frequencies of rs71212732 (T\C) polymorphism and both disease severity and improvement after treatment.

Chen et al., found significantly increased level of (CD100) on keratinocytes of psoriatic skin, but he also found the expression of Plexin B2 in normal skin of psoriasis patients was similar to that of healthy individuals [18].

In their report, Sinha et al.,found that Multiple genome-wide association studies (GWAS) studies have shown that the SNP rs2281808 TT variant, present within the SIRPG (signal regulatory protein gamma) gene, is associated with autoimmune diseases, such as type 1 diabetes [19].

In the present study, we investigated SIRPG genotypes and their effects on the fate and function of human T-cells. We found that the presence of T allel variant resulted in reduction of SIRPγ expression on T-cells. Functionally, SIRPγ low CD8 T-cells in CT and TT individuals existed in a heightened effector state with lower activation threshold and had greater expression of genes and molecules associated with migratory and cytotoxic potential. Further, SIRPγ low CD8 T-cells were deficient in transcription factors associated with long-term functional memory formation. Our study reveals biological consequences of the SNP rs2281808 and provides novel insights into the potential mechanisms by which SIRPγ might regulate human immune responses**.**

Smith et al., agreed with our study and found that SIRPG is the most likely causative gene for type 1 diabetes risk in the 20p13 region and highlight the role of alternative splicing in lymphocytes in mediating the genetic risk for autoimmunity. However, little is known about its role in skin diseases. Activation of Plexin-B2 may play a role in the pathogenesis of psoriasis [20]. Zhang et al., tried to investigate the role of plexin-B2 in the pathogenesis of psoriasis and suggested that it may be used as a therapeutic target in psoriasis. Confirming its role in activating keratinocytes proliferation [21], Witherden et al.,reported that plexin-B2 is expressed in normal mouse keratinocytes and is involved in keratinocyte proliferation and the repair process in wound healing and tissue maintenance [5].

There was significant overexpression of plexin-B2 in dermal inflammatory cells of lesional skin of psoriasis patients when compared to controls skin. Kolodkin, described that plexin-B2 receptors serve important roles in T cell priming, antibody production, and cell-to-cell adhesion [22]. In addition, Yan et al., found that the interaction of plexin-B2 and its ligand, CD100, can stimulate T cell activation and function in the germinal center. These data support the inflammation-promoting role of plexin B in psoriasis [23].

The present study suggested that tissue plexin-B2 level has a good predictive power with clinical correlation for marked improvement, the validity criteria were as follows; 73% sensitivity which means that tissue Plexin B2 correctly identified 73% of positive cases as having marked improvement. Also, 69% specificity, the test correctly identified 69% of those without marked improvement as negative. Additionally, the test had 70% precision—Positive Predictive Value (PPV) i.e., the ability of the test to predict actively diseased patients among all positive cases. It also had 72% Negative Predictive Value (NPV) i.e., the ability to predict those with inactive disease among all those diagnosed as negative and overall, the test had 71% accuracy and also tissue plexin B2 level had a good predictive power for marked improvement. The validity criteria were as follows; **78% **sensitivity, i.e., tissue plexin B2 correctly identified 78% of positive cases as having sever disease. Also, **73%** specificity, additionally, the test had 74% precision—Positive Predictive Value (PPV) i.e., the ability of the test to predict actively diseased patients among all positive cases. It also had 77% Negative Predictive Value (NPV) i.e., the ability to predict those with inactive disease among all those diagnosed as negative and overall, the test had 75.5% accuracy.

## Conclusion

The present study concluded that tissue plexin B2 is higher in psoriasis than normal and positively correlated with disease severity. Tissue plexin B2 was significantly reduced after treatment with significant reduction in combined than single treatment. There was significant difference between cases and controls in genotype frequency distribution (TT/TC/CC), wild homozygous (TT) noticed markedly in control group, mutant heterozygous (TC) and mutant homozygous (CC) noticed markedly in cases group. These suggest that tissue plexin B2 may have a role in pathogenesis of psoriasis and can be a good marker for disease progress and improvement.

## Limitations

The main limitation of this study that we could not account for other clinical factors associated with tissue Plexin–B2 levels variations & related gene polymorphism. Also other causes of variations in Plexin–B2 not considered.

## Data Availability

The data that support the findings of this study are available on request from the corresponding author, [Hegazy, EM]. The data are not publicly available due to [they are containing information that could compromise the privacy of research participants].
